# Digital Competence of Tourism Students: Explanatory Power of Professional Training

**DOI:** 10.3390/ejihpe10010024

**Published:** 2019-12-16

**Authors:** Ambar J. Arango-Morales, Alejandro Delgado-Cruz, Ana L. Tamayo-Salcedo

**Affiliations:** Facultad de Turismo y Gastronomía, Universidad Autónoma del Estado de México, Toluca de Lerdo 50000, Mexico; ajarangom@uaemex.mx (A.J.A.-M.);

**Keywords:** digital competence, professional training, tourism students

## Abstract

This article aims to analyze the influence of professional training on the digital competence of tourism students at a Mexican public university. For this, a quantitative methodology was used through the application of a survey amongst 400 students. Moreover, the partial least squares structural equation modeling (PLS-SEM) and other multivariate techniques were used to test the hypotheses. The results show that the role of teaching, the curriculum proposal, and the student autonomy as part of professional training all have an impact on digital competence in order to generate and effectively use digital knowledge, manage information in support of their activities and use the media in individual and collective channels, as well as emancipate collaborative learning and maintain leadership in the network. It is discussed how the emphasis of curricular programs and the support of teachers play a significant role as cultivators of digital competence, as well as the initiative and independence of students to better exploit digital media for their personal, academic and professional activities.

## 1. Introduction

The digital transformation experienced worldwide directly affects the development of society, mainly students in full professional training who will soon enter the work field. Therefore, professional training for the acquisition and integration of digital knowledge, skills, and abilities is a necessity for any individual immersed in productive activities [1,2,3,4,5]. At the same time, this training becomes a great challenge for higher education institutions, which includes the development of critical thinking, effective communication, problem-solving and decision making through the management and exploitation of technology [6,7,8].

Despite the fact that new generations of students have a native technological confidence when interacting daily with mobile devices, platforms, and social networks, there are problems linked to digital competence as their capability of attention, communication, and learning to deal with problem solving is limited, compared to those generations that grew up in contexts with low mediation of digital technologies [3].

Faced with this, researchers, academics, and stakeholders in higher education have paid special attention to the study of digital competence as a means of enabling individuals to experience more satisfying and productive personal, academic and professional lives [3,4]. For example, Almerich et al. [9] point out that students will have to acquire digital skills to adapt to a society characterized by the fusion of digital, physical, and even biological technologies.

Literature reveals that digital competence goes beyond technical connotation, since the way a person thinks, solves problems, and learns has a greater impact on his or her adaptation and development in the technological context, as opposed to a person who only has operative management of some specific software or technology [10,11,12]. Thus, digital competence integrates digital knowledge, skills and abilities in the domain of technologies along with higher-order cognitive processes that favor continuous learning.

In the context of education and recognition of the potential benefits of emerging technologies and digital environments, schools have been called upon to integrate digital platforms and tools to support teaching and learning systems [3,4,13]. However, problems related to professional training have come into play, such as the lack of connection between the emphasis of curricular programs and teacher support, which leads to a barrier to the development of digital competence in students [2,7,14]. Likewise, the limited integration of digital technologies and media as pedagogical support tools, the resistance of the students to take advantage of mobile devices, and the lack of autonomy to be trained in their use are reported [15].

In tourism, as well as in other sectors of economic relevance, digital competition is a mandatory 21st-century requirement to achieve innovation [1,12,16]. The works show that as digitalization advances in the field of tourism, management processes in organizations, destinations, and the labor force change, as well as the needs of tourists and residents [4,17,18,19]. It is, therefore, demanded that decision-makers, government representatives, business managers, and service providers be prepared to meet the demands of the tourism market through digital competition [4,16,20]. 

In this sense, tourism education intervenes in the professional training of the new actors that will guide the course of the sector [15,21], and digital competition could be a strategic asset to compete in a highly demanding market. Nonetheless, empirical work and available frameworks are still incipient to provide operational components that help educational institutions to better target their actions in the training of students [12]. For this reason, this paper aims to analyze the influence of professional training on the digital competence of tourism students at a Mexican public university. To account for this, it first presents the fundamentals that support the research hypotheses. The methodology followed in the collection and analysis of data is clarified to give way to analysis and discussion of results. Finally, conclusions, future lines of research, and limitations of the work are presented.

## 2. Literature Review

Digital competence is associated with the goals, expectations, and workforce of knowledge citizenship [14,22]. Within this framework, the conceptualization of digital competence has had a long-term development, and its contemporary aspect is characterized by its complexity, not only in technical skills but also in cognitive and attitudinal components [23]. Besides, it has become a key term in the discussion about what kind of skills, knowledge, and aptitudes people should have in the knowledge society, reflecting beliefs and desires about future needs, as well as thinking about new technologies as opportunities and solutions to economic contingency.

The problem with the conceptualization of digital competence is that in the scientific literature there is no consensus on what it is. On the contrary, it presents a complex range of interrelated visions [10,11,12]. Some authors have succeeded in articulating the domains of learning, the tools and the purposes that reflect the essence of digital competence. For example, Ilomäki et al. [22] envision that digital competence is the ability to use digital technologies meaningfully in everyday life, leading to a commitment to participate actively in digital culture. While Cahen and Borini [24], and Ferrari et al. [25] consider it a combination of knowledge, talents, skills, and attitudes linked to the use of technology to perform tasks, solve problems, communicate, and manage information. Adding to this, a critical, ethical, and responsible behavior when collaborating, creating, and sharing digital content for work, leisure, and social participation.

In this vein, the practicality of digital competence can be seen in various contexts of personal and professional life [3,26,27]. In the education context, Guzmán-Simón el al. [2] point out that digital competence should focus on individual skills and the ability of the academic institution to integrate people into cultural and social practice. Educational institutions must, therefore, become spaces governed by creativity, discovery, and digital navigation in support of the intellectual evolution of students [14].

The direct immersion of technology in the field of work represents a challenge for educational institutions since it is necessary to train young university students in skills to meet the digital demands of society. Based on the contributions of different authors, it can be said that digital competence integrates: Digital knowledge, information management, individual and collective digital communication, collaborative learning, and networked leadership [10,12,18,22,24,25,27]. Therefore, professional training is imposed in terms of a complete understanding of the technological phenomenon and the use of digital media to enable students to develop knowledge, skills, and abilities that allow them to do their jobs effectively.

In particular, the tourism sector is characterized by the intensive use of information and communication technologies (ICT) in the provision of services and by serving a market of consumers highly dependent on these technologies to seek and acquire the best options for services and vacation destinations. Faced with this situation, it is necessary to have people trained in technologies to develop digital content and tourism products, operate business models mediated by digital platforms, and implement digital marketing strategies [16,28]. For this reason, the tourist workforce is related to the professional training that students receive, who, in the future, will become decision-makers and strategists in this sector [15,16,17,20,21].

For its part, professional training can be seen as a set of teachings whose purpose is to train people to perform any professional activity involving elements such as: The role of the teacher or trainer, the curriculum proposal, and the student autonomy [10,18,22,27]. The teaching role as a component of professional training intervenes in the development of digital competence, as it involves a series of activities associated with student motivation in their learning processes. It is also recognized that teachers, through their pedagogical skills, didactic judgment, and awareness of the strategic implications of learning, are facilitators of useful knowledge for the working practice of students [6,7,11].

On the other hand, the curriculum proposal makes it possible to articulate the curricula and content programs of classes with the development of competencies inside and outside the classroom to be successful. For this reason, the curriculum proposal should preferably be formulated based on educational policy documents at the different levels of the system, that is, at the institutional, regional, and national levels [10,18,22,27]. Otherwise, the degree of digital competence and its articulation with social policies and needs could lead to educational and employability recessions [10].

Student autonomy also becomes a key element in triggering digital competence. Today, despite easy access to digital resources and media, there is no guarantee that individuals will automatically have the digital competence to take full advantage of them [15]. Thus, the academic commitment and initiatives undertaken by students fit with their professional performance and the development of digital competence by generating self-learning mechanisms. Thus, students who do not have solid digital literacy may face low academic performance and fewer employment opportunities in a competitively employed sector [5].

## 3. Methodology

### 3.1. Research Hypotheses

As digital technologies are becoming a central part of daily work, institutions are increasingly forced to rethink and transform their educational practices to contribute to the professional training of tourism students who, in the future, will be inserted into the labor field. Because of this, the following research hypotheses are put forward:

H_1a_ = There is a significant and positive relationship between the teaching role and the dimensions of digital competence (digital knowledge, information management, individual digital communication, collective digital communication, networked collaborative learning, and network leadership).

H_1b_ = There is a significant and positive relationship between the curriculum proposal and the dimensions of digital competence (digital knowledge, information management, individual digital communication, collective digital communication, networked collaborative learning, and network leadership).

H_1c_ = There is a significant and positive relationship between student autonomy and the dimensions of the digital competence (digital knowledge, information management, individual digital communication, collective digital communication, networked collaborative learning, and network leadership).

H_2_ = Professional training has a significant and positive influence on the digital competence of tourism students.

### 3.2. Research Design

This research work conforms to its construction logic under the post-positivist viewpoint, and the hypothetical-deductive method by testing prepositions sketched from theory with the support of measurement and statistical analysis of phenomena [29]. It is a cross-sectional and of non-experimental design by collecting the data at one time and presenting the situation as it is being observed. Likewise, the scope is explanatory since it not only sought to relate the observed variables but also to explain why and in what conditions professional training influences digital competence [30].

### 3.3. Target Population and Sample

The context of the study was the Universidad Autónoma del Estado de México, in the city of Toluca, Mexico. In particular, it was limited to the Faculty of Tourism and Gastronomy recognized nationally and internationally for its historical trajectory to be a pioneer in the formation of tourism professionals in Latin America. Therefore, it was a population of 682 students of the bachelor of tourism of this academic space [31], and when considering the goodness of fit for the validity of the model, it opted for a non-probabilistic sampling and a selection technique by convenience subjects, thus forming a sample of 400 students. It is recognized that the participating students belong to the same school where the researchers work.

As shown in Table 1, the research sample was tourism students from a Mexican public university. Of the sample, 73.5% were women, and 26.5% were men, unmarried youth between 18 and 22 years of age (97.7%). Most were residents of the city of Toluca (53.75%) and the municipalities of Metepec, Zinacantepec, San Mateo Atenco, and others (26.5%). According to their academic progress, the students were in the following school years: first (35.2%), second (26.5%), and fourth (19.2%). As for their employment situation, 19.75% of the students have a job and half of them are in the tourism industry (53.16%). Regarding their economy, students spend between $40 and $100 Mexican pesos (equivalent to two and five U.S. dollars) per week for school supplies, and 78.3% use public transportation and 15.5% walk to school, reflecting a low-medium economic status.

### 3.4. Data Collection

The technique for data collection was a self-administered survey. For this, students were personally invited during class and break times. The instrument was provided by the applicators, who gave the instructions to complete it. In this process, the participants were informed in writing about the academic use of the data and the guarantee of confidentiality of their answers. The application of the survey took place during March 2019, an opportune moment since students from all grades are in the school for the delivery of papers and evaluations, which increased the possibilities of participation.

### 3.5. Research Instrument 

The construction process of the items and design of the research instrument was based on the following stages: an exhaustive review of the literature, validity of content by experts in the field, pilot test, pilot re-test, and statistical evaluation of its reliability and validity. For the identification of the constructs, the exploratory factorial analysis (EFA) was applied by main components, in which nine dimensions were considered with an explained variance of 66.58%. Therefore, the dimensions of professional training as an independent variable and digital competence as a dependent variable were conceptually and operationally defined, as shown in Table 2 and Appendix A.

Thus, the instrument consisted of two sections. The first contained the items to measure the variables in question (Table A1), which were evaluated with a Likert-scale scale where 1 was “totally disagreed”, 2 “moderately disagreed”, 3 “slightly disagreed”, 4 “slightly agreed”, 5 “moderately agreed” and 6 “totally agreed”. The second section of the instrument consisted of a technical sheet to obtain sociodemographic data on respondents (Table 1).

### 3.6. Data Analysis

By corroborating the non-parametric distribution of the data with the symmetry and kurtosis values of some items and constructs (Table 3), the central technique for data processing was the modeling of structural equations by partial least squares (PLS-SEM) for its explanatory capability of empirical verification of the theory with non-parametric data [33]. Bivariate correlation using the Spearman coefficient (r) was also used to analyze the degree of relationship between the dimensions of professional training and digital competence. Central tendency and dispersion measurements were used to describe the sample and its appreciations. Data processing was supported by two statistical packages: SPSS version 25 [34] and Smart PLS version 3 [35].

## 4. Results

### 4.1. Descriptive Analysis

Table 3 shows the mean and standard deviations of the constructs. Student autonomy ($\tilde{x}=\;$5.240, σ = 0.744) was the most appreciated dimension of professional training, although the teaching role and the curriculum proposal have positive values, but at a low level, according to the measurement scale. In particular, students are engaged academically (SA_01) in seeking alternatives to improve their training (SA_02), with the support of technological resources (SA_03), the application of knowledge acquired in school (SA_04) and independent study (SA_06). As for the curriculum proposal, there is an interest in the avant-garde (PC_06), the clarity of the aims (PC_01) and the attention to the demands of the economic sectors (PC_02) that the educational program considers, as well as the usefulness of the content of the classes for the application of knowledge (PC_07), decision making (PC_10) and problem solving (PC_09). While in the teaching role, the experience of teachers in the field of tourism (TR_06) and their ability to demonstrate the usefulness of knowledge in real contexts stand out (TR_07). 

Similarly, the dimensions of digital competence are prescribed as positive and low, except for information management ($\tilde{x}\;$= 5.260, σ = 0.642) and individual digital communication ($\tilde{x}$ = 5.276, σ = 0.706) which reach a medium level (Table 3). In the digital competence, the following stand out: a) The application of knowledge on digital resources to facilitate academic, work and family activities (DK_04), b) the location (IM_01), obtaining (IM_02) and responsible use (IM_04) of reliable information (IM_03), and c) the establishment of digital communication channels (IDC_03) quickly (IDC_02) and efficiently (IDC_01). However, the values of collective or team communication are low, suggesting that little value is generated to solve problems when participating in conversations or debates (CDC_01). 

In general terms, it is diagnosed that these university students perceive as sufficient both the professional training they receive from the institution and the digital competence they have developed, but without reaching levels of excellence.

### 4.2. Correlational Analysis

As expected, the relationships between the dimensions of professional training: teaching role, curriculum proposal, and student autonomy were highly significant (Table 4). Among these, the association of the teaching role with the curriculum proposal stands out because it is moderate-strong (r = 0.693, *p* < 0.010), showing that teaching skills, the use of pedagogical methods, and the professional experience of teachers as facilitators of knowledge support the effective management of the curricular proposal. Therefore, it is appreciated for having clear objectives and remaining at the forefront of tourism education, thus helping students to be better decision-makers and problem-solving strategists.

Moreover, between the dimensions of digital competence (digital knowledge, information management, individual digital communication, collective digital communication, networked collaborative learning, and network leadership) highly significant correlations were presented, ranging from weak-moderate to moderate. The associations of networked collaborative learning with network leadership (r = 0.613, *p* < 0.010) and collective digital communication (r = 0.521, *p* < 0.010) stand out, revealing that shared experience facilitates the direction and influence that leaders have over their work teams, as well as speeding up the communicative process in academic and professional tasks and activities.

Regarding the crossing of variables, it was found that the teaching role is linked to the dimensions of digital competencies, except with collective digital communication (r = 0.093, *p* > 0.050). Reflecting that the teaching skills of the teacher do not intervene in the development of communication competence in collectives in the students, inferring that this may be due to autonomous practice. However, the teaching role is weakly related to digital knowledge (r = 0.350, *p* < 0.010) and information management (r = 0.305, *p* < 0.010). Therefore, it is shown that the intellectual competencies to search for, evaluate, and use information, as well as the knowledge and adaptation that students have in digital environments, corresponds to the teacher’s guide. With such results, the H_1a_ hypothesis is partially accepted.

The results show that the curriculum proposal has links with digital knowledge (r = 0.435, *p* < 0.010), network leadership (r = 0.458, *p* < 0.010) and networked collaborative learning (r = 0.427, *p* < 0.010). In this sense, the perceived content of the educational program meets the current demands of the tourism sector and is involved in the development of leadership skills to be aware of digital changes and contribute to the learning of others. In this way, support is given to accept the _H1b_ hypothesis.

Concerning student autonomy, it was observed that it has highly significant and positive relationships with the dimensions of digital competence. In particular, the associations with information management (r = 0.435, *p* < 0.010), digital knowledge (r = 0.458, *p* < 0.010), networked collaborative learning (r = 0.427, *p* < 0.010) and network leadership (r = 0.496, *p* < 0.010) are striking. Therefore, it can be said that the initiative and commitment of the student in his or her training is linked to digital competence by seeking new ways of using technological means, managing digital information, and learning on the net to develop in tourism, results that allow the H_1c_ hypothesis to be tested.

### 4.3. Explanatory Analysis

**Figure 1:**
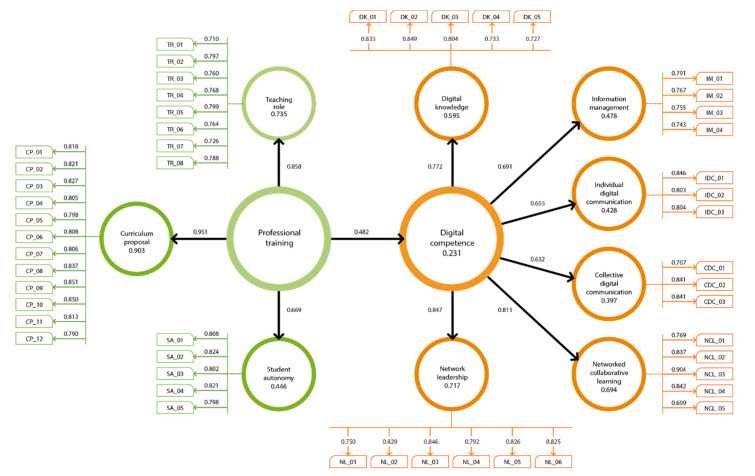
Explanatory model for professional training on digital competence.

For the measurement model, the values of internal consistency (α), composite reliability (CR), and rho_A for each construct were satisfactory at > 0.700 and CR > AVE [33] (Table 5). Likewise, the convergent and discriminant validity was fulfilled (Table 6), the first by obtaining values of the average variance extracted (AVE) greater than 0.500 and the second by comparing the value of the square root of the AVE with the correlation between the constructs according to estimates by Fornell and Larcker [36]. Finally, Figure 1 shows the loads for each item, which are close to or greater than the recommended value (>0.700). Considering such values, the reliability, and validity of the constructs of the proposed model can be guaranteed.

To assure the fit and validity of the structural model, the Bootstrapping function was used with a total of 5000 cases [37] to verify the mean square residual root (SRMR) [38], which was 0.101. In addition, the values of t and significance were considered for each of the relationships, which meet the criteria t > 1.96 and *p* < 0.000 (Table 7).

In Figure 1, it can be seen that professional training explains 48% of the variance of digital competence (γ = 0.482, *p* < 0.010) and assumes a predictive power of 23% (R^2^ = 0.233, *p* < 0.010). Likewise, the indirect effects of professional training trajectory on the dimensions of digital competence with low-moderate impact are observed, particularly with networked collaborative learning and network leadership (Table 8). With these results, the central research hypothesis (H_2_) is supported and it can be affirmed that the teaching role, the curriculum proposal, and the student autonomy as elements of professional training have an impact on digital competence to creatively create and use digital knowledge, manage information in support of academic activities, effectively use the media, and emancipate collaborative learning and leadership in the network.

## 5. Discussion

To the extent that the content and nature of the works are changed by the phenomenon of digitization, so is the competence required to carry them out. This shapes the patterns of employment and professional training offered in educational institutions. In the context of tourism education, this research shows that both professional training and digital competence are evaluated positively but at low levels. This can be explained by Porat et al. [3], who point out that students, despite living in a context mediated by technologies and having a greater facility to acquire and manipulate them, do not necessarily exploit them to solve problems.

In spite of this, it cannot be concluded that there is a digital gap, since among the most highly valued dimensions are student autonomy, information management, and individual digital communication, which account for sufficiently independent students who take advantage of Internet tools to search for, transmit and use information responsibly in their daily work [7,8]. Based on the contributions of Adukaite et al. [15], this research also demonstrates that students’ autonomy allows them to use digital media and to make effective use of the knowledge acquired in the classroom in their personal and professional activities.

On the other hand, the emphasis on curricular programs and the support of teachers play a significant role in fostering digital competence. Thus, the set of teachings, pedagogical methods, and professional experience of teachers help in the implementation of curricula and classes, while equipping students with the digital competence that will affect their work performance [5,32]. Consequently, educational institutions must have mechanisms to train teachers in technology from a holistic and non-reductionist perspective, that is, to foresee that technological use in education and the professional field includes the development of disruptive, reflective and critical thinking for the creative and effective solution of problems.

In coincidence with Pettersson [10], it can be seen that curricula and class contents, when mediated by technologies, enrich the teaching-learning process to a greater degree. At the same time, their success depends on the postulation of clear objectives and their articulation with educational policies. Therefore, attention should be paid to the design of curriculum proposals to help students confront emerging pedagogies and adjust their level of knowledge in technological matters [6].

Similarly, it is stated that the curriculum proposal is associated with digital knowledge, network leadership, and networked collaborative learning, reflecting that this educational channel should be focused on providing students with the technological tools to develop their capability to adapt to the tourist environment. Therefore, future studies must address this approach to know whether computer type, Internet access, smartphone capabilities, online platforms. and library resources are involved in the development of digital competence.

In convergence with Pettersson [10], Spante et al. [11], and Van Laar et al. [12], the findings show that digital competence does not stagnate in an operational skill, but is also accompanied by a set of knowledge, skills, and attitudes to provide solutions to everyday problems and make assertive decisions in a technological context. Based on the predictive power found and the work of Almerich et al. [9], it can be said that, in the long term, the combination between professional training and digital competence allows for the development of better adaptation capabilities and learning mechanisms, as well as increasing the academic performance and motivation of students.

Because of the above, students of tourism must continuously update their digital competence to face the labor market. At the same time, emphasizing their willingness to efficiently exploit the knowledge that is provided during their professional preparation, otherwise, their chances of successfully inserting themselves in productive activities will be diminished.

## 6. Conclusions

The accelerated pace and rapid integration of technologies into the current environment make it essential to acquire the digital competence necessary for employment and participation in society. In particular, it is relevant to understand how digital knowledge, skills, and abilities are developed from different aspects, including professional training. Because of this and in compliance with the research aim, this empirical study confirms that professional training has significant explanatory power over the digital competence of tourism students.

Through the results, it is evident that tourism students do not have a high level of digital competence and, therefore, it is essential to design and develop training and accreditation processes that allow the level of this competence to be evidenced. Likewise, it can be concluded that the teaching role increases the possibilities for students to generate, acquire, and use the knowledge that allows them to develop professionally and personally in the digital economy. In the same way, when the curriculum proposal is aligned with the demands of employers and the vanguard of the tourism sector, leadership and collaborative learning are favored. For the student autonomy, this is essential to expand their academic preparation, manage information, use digital knowledge, and learn collaboratively in technological platforms, desirable characteristics for a high professional profile in tourism.

Considering that the environment is highly digital and educational institutions have the responsibility of enabling students to enter the labor market, the practical implication of this research proposes to determine what level of digital competence they have when they arrive at the university and what it will be at the end of their professional studies to ensure their participation in economic activities and thus articulate institutional efforts towards this end.

In terms of future research lines, it would be interesting to triangulate the results from other perspectives, for example, from teachers and employers. The former are responsible for directing the professional training of students and the latter for their ability to assess digital competence in work practice. The studies would have to take up variables linked to professional training, such as educational policy, the quality of technological infrastructure and the technological expertise of teachers. Other lines could address the relationship of digital competition with job opportunities and professional projection in the tourism sector, as well as its intervention in the innovation of tourism products mediated by technologies.

Future research should also include a replication study with other schools from different countries, particularly those dependent on tourism. Cultural, social, and economic factors could intervene in the digital competence of students and professionals, such as the level of development of the nation, public policies, government support for education, the participation of the business sector, among others. It would be a contribution to knowledge to identify which technologies are most used by students for their school and professional activities, as well as to know how they intervene in the development of digital competence.

Finally, on of the limitations of the work is the sample, since it was a group of tourism students of a Mexican university. Therefore, the results cannot be generalized because significant changes could exist if other educational entities in the branch were considered. In this sense, it is suggested to integrate samples of students belonging to other public and private institutions that offer tourism careers at the national and international levels. In addition, it is recommended to carry out confirmatory statistical analyses when using more robust techniques, such as the covariance-based structural equation modeling (CB-SEM).

## Figures and Tables

**Table 1:** Sample description.

Variable	Value	Percentage
Gender	Masculine	73.5%
	Feminine	26.5%
Age	16–20	62.4%
	21–25	35.3%
	26–30	2.7%
Academic year	1	35.3%
	2	26.5%
	3	12.8%
	4	19.3%
	5	5.3%
	Other	1%
Birthplace	State of Mexico	78.8%
	Other federative entity of Mexico	19.2%
	Foreign country	2.8%
Place of residence	Toluca	53.8%
	Metepec	8.5%
	Zinancatepec	6%
	San Mateo Atenco	5.3%
	Lerma	3.3%
	Santiago Tianguistenco	3.3%
	Ciudad de México	2.5%
	Other	18.6%
Marital status	Single	97.3%
	Free union	1.8%
	Married	0.8%
	Divorced	0.3%
Job	Yes	57.3%
	No	42.5%
Job related to tourism activity	Yes	53.16%
	No	46.8%
Transportation to school	Public transport	63.7%
	Walking	15.5%
	School transportation	7%
	Family car	6.8%
	Own car	6.5%
	Other	0.5%
Expenditure on school material per week (in Mexican pesos)	$40–$100	78.3%
	$101–$200	23.8%
	$201–$300	10.5%
	More than $301	10.8%

**Table 2:** Conceptual definition of variables.

Variable	Dimension
**Professional training (PT):** A set of teachings whose purpose is to enable people to carry out a professional activity [10,18,22,27].	**Teaching role (TR):** The role of the teacher as a facilitator of useful knowledge for professional practice, a pillar of inspiration, and a promoter of educational quality through experience, performance, teaching methods, and updating [8].
	**Curriculum proposal (CP):** A set of pedagogical elements that are embodied in curricula and class content that focus on strengthening skills inside and outside the classroom to be successful [10,18,22,27].
	**Student autonomy (SA):** Students’ attitude and initiative to exploit their professional training independently as a complement to the education they receive in the classroom [5,15].
**Digital competence (DC):** Combination of knowledge, skills, talents, and attitudes linked to the use of technology to perform tasks, solve problems, communicate and manage information. Adding to this, critical, ethical, and responsible behavior in collaborating, creating, and sharing digital content for work, leisure, and social participation [22,24,25].	**Digital knowledge (DK):** A set of skills for professional and personal development in the digital economy [22,24,25].
	**Information management (IM):** The ability to search for, obtain, evaluate, organize and share the most appropriate information through ICTs to respond to a given task [12,18,25].
	**Individual digital communication (IDC):** Intrapersonal ability to communicate efficiently and effectively with digital tools [12,26].
	**Collective digital communication (CDC):** Interpersonal ability to collaborate with others in communication processes efficiently and effectively with digital tools [12,18,26].
	**Networked collaborative learning (NCL):** Learning capability in work teams to acquire knowledge and experiences that strengthen the effective use of digital media [12,32].
	**Network leadership (NL):** Ability to influence, coordinate, and lead work teams distributed in the network and digital environments [18,19].

**Table 3:** Descriptive statistics of items and constructs.

Item	Mean	Standard Deviation	Asymmetry	Kurtosis	Construct	Mean	Standard Deviation	Asymmetry	Kurtosis
TR_01	4.840	0.952	−0.778	0.956	TR	4.837	0.788	−1.042	1.559
TR_02	4.800	0.965	−1.068	1.803					
TR_03	4.720	1.007	−0.659	0.282					
TR_04	4.525	1.061	−0.666	0.570					
TR_05	4.895	1.073	−0.988	0.962					
TR_06	5.055	1.004	−0.975	0.520					
TR_07	5.060	0.994	−1.243	1.754					
TR_08	4.805	1.185	−1.105	0.992					
CP_01	4.530	1.152	−0.839	0.521	CP	4.575	0.969	−1.068	1.039
CP_02	4.657	1.163	−0.937	0.708					
CP_03	4.375	1.246	−0.732	0.184					
CP_04	4.377	1.218	−0.821	0.435					
CP_05	4.367	1.357	−0.785	−0.063					
CP_06	4.502	1.211	−0.774	0.315					
CP_07	4.705	1.100	−0.756	0.028					
CP_08	4.682	1.074	−0.657	0.091					
CP_09	4.642	1.110	−0.805	0.255					
CP_10	4.760	1.169	−0.969	0.662					
CP_11	4.760	1.241	−1.124	0.988					
CP_12	4.545	1.177	−0.817	0.345					
SA_01	5.380	0.915	−1.771	3.769	SA	5.240	0.744	−1.529	3.276
SA_02	5.032	0.947	−1.094	1.579					
SA_03	5.312	0.872	−1.380	2.100					
SA_04	5.277	0.895	−1.269	1.586					
SA_05	5.197	0.959	−1.276	1.773					
DK_01	4.822	0.942	−0.795	0.899	DK	4.847	0.726	−0.798	0.905
DK_02	4.840	0.886	−0.678	0.776					
DK_03	4.827	0.888	−0.646	0.707					
DK_04	5.292	0.783	−1.068	1.229					
DK_05	4.452	1.104	−0.596	0.200					
IM_01	5.475	0.731	−1.474	2.494	IM	5.260	0.642	−1.319	3.421
IM_02	5.235	0.875	−1.084	1.050					
IM_03	5.037	0.968	−1.006	1.398					
IM_04	5.295	0.780	−1.077	1.772					
IDC_01	5.410	0.783	−1.398	2.164	IDC	5.276	0.706	−1.184	1.500
IDC_02	5.397	0.866	−1.561	2.343					
IDC_03	5.022	0.932	−0.865	0.732					
CDC_01	3.712	1.369	−0.324	−0.596	CDC	4.310	0.983	−0.747	0.362
CDC_02	4.450	1.149	−0.703	0.294					
CDC_03	4.767	1.184	−1.025	0.889					
NCL_01	4.737	1.082	−0.846	0.620	NCL	4.662	0.904	−0.748	0.351
NCL_02	4.657	1.112	−0.807	0.626					
NCL_03	4.660	1.150	−0.868	0.651					
NCL_04	4.800	1.099	−0.895	0.542					
NCL_05	4.457	1.134	−0.573	0.098					
NL_01	4.647	1.123	−0.820	0.644	NL	4.847	0.835	−0.716	0.289
NL_02	4.792	1.064	−0.870	0.585					
NL_03	4.880	1.009	−0.787	0.292					
NL_04	4.935	0.989	−0.806	0.204					
NL_05	4.947	0.952	−0.875	0.856					
NL_06	4.882	1.047	−0.882	0.663					

**Table 4:** Spearman correlations coefficients.

Dimension	TR	CP	SA	KD	IM	IDC	CDC	NCL	NL
Teaching role (TR)	1								
Curriculum proposal (CP)	0.693 **	1							
Student autonomy (SA)	0.447 **	0.500 **	1						
Digital knowledge (DK)	0.350 **	0.319 **	0.458 **	1					
Information management (IM)	0.305 **	0.252 **	0.435 **	0.483 **	1				
Individual digital communication (IDC)	0.250 **	0.220 **	0.307 **	0.356 **	0.410 **	1			
Collective digital communication (CDC)	0.093	0.197 **	0.293 **	0.355 **	0.258 **	0.320 **	1		
Networked collaborative learning (NCL)	0.291 **	0.336 **	0.427 **	0.477 **	0.391 **	0.430 **	0.521 **	1	
Network leadership (NL)	0.320 **	0.326 **	0.496 **	0.502 **	0.491 **	0.363 **	0.450 **	0.613 **	1

Note: ** The correlation is highly significant at level 0.01 (bilateral).

**Table 5:** Reliability of constructs.

Dimension	α	CR	rho_A	AVE
Teaching role (TR)	0.898	0.918	0.899	0.585
Curriculum proposal (CP)	0.955	0.961	0.956	0.670
Student autonomy (SA)	0.870	0.906	0.874	0.657
Digital knowledge (DK)	0.849	0.892	0.851	0.625
Information management (IM)	0.763	0.849	0.764	0.584
Individual digital communication (IDC)	0.756	0.858	0.773	0.669
Collective digital communication (CDC)	0.715	0.840	0.733	0.638
Networked collaborative learning (NCL)	0.871	0.904	0.877	0.613
Network leadership (NL)	0.896	0.921	0.898	0.659

**Table 6:** Validity of constructs.

Dimension	TR	CP	SA	DK	IM	IDC	CDC	NCL	NL
Teaching role (TR)	0.765 *								
Curriculum proposal (CP)	0.730	0.819 *							
Student autonomy (SA)	0.456	0.493	0.811 *						
Digital knowledge (DK)	0.364	0.356	0.499	0.791 *					
Information management (IM)	0.298	0.280	0.506	0.536	0.764 *				
Individual digital communication (IDC)	0.253	0.227	0.412	0.412	0.480	0.818 *			
Collective digital communication (CDC)	0.079	0.151	0.286	0.348	0.262	0.387	0.799 *		
Networked collaborative learning (NCL)	0.282	0.324	0.504	0.515	0.434	0.515	0.568	0.783 *	
Network leadership (NL)	0.287	0.314	0.519	0.555	0.505	0.412	0.466	0.620	0.812 *

Note: * Square root value of the average variance extracted (AVE).

**Table 7:** Path coefficients.

Dynamic	Original Sample	Sample Mean	Standard Deviation	t-Value	Sig	R^2^	R^2^_aj_
PT→DC	0.482	0.482	0.056	8.594	0.000	0.233	0.231
PT→TR	0.858	0.858	0.018	46.975	0.000	0.736	0.735
PT→CP	0.669	0.670	0.041	16.180	0.000	0.904	0.903
PT→SA	0.951	0.951	0.006	154.940	0.000	0.447	0.446
DC→DK	0.772	0.772	0.026	29.654	0.000	0.596	0.595
DC→IM	0.692	0.692	0.039	17.608	0.000	0.479	0.478
DC→IDC	0.655	0.655	0.039	16.715	0.000	0.430	0.428
DC→CDC	0.631	0.633	0.034	18.397	0.000	0.398	0.397
DC→NCL	0.833	0.834	0.020	42.615	0.000	0.694	0.694
DC→NL	0.847	0.848	0.016	53.641	0.000	0.718	0.717

**Table 8:** Indirect effects.

Dynamic	Original Sample	Sample Mean	Standard Deviation	t-Value	Sig
PT→DK	0.372	0.373	0.051	7.328	0.000
PT→IM	0.334	0.334	0.049	6.750	0.000
PT→IDC	0.316	0.316	0.046	6.857	0.000
PT→CDC	0.304	0.305	0.038	7.972	0.000
PT→NCL	0.402	0.402	0.049	8.200	0.000
PT→NL	0.409	0.409	0.049	8.288	0.000

## Appendix A

**Table A1:** Operational definition of variables.

Variable	Dimension	Code	Items
Professional training (PT)	Teaching role (TR)	TR_01	Teachers act as facilitators of useful knowledge for my professional training
		TR_02	Teachers show teaching skills that affect my professional development
		TR_03	Teachers promote in me the development of professional competencies
		TR_04	Teachers use appropriate methods to assess my professional development
		TR_05	Teachers are up to date on the topics of the tourism context that influence my training
		TR_06	Teachers have professional experience in the field of tourism that strengthens my training
		TR_07	Teachers use real cases to show their usefulness in the professional field
		TR_08	Teachers inspire you to be a better tourism professional
	Curriculum proposal (CP)	CP_01	The syllabus contemplates clear objectives for me to be formed as a professional of the tourism
		CP_02	The syllabus identifies the sectors or fields where I can develop professionally
		CP_03	The syllabus considers learning units with high applicability in the professional context
		CP_04	The syllabus is at the forefront in relation to the demands of the professional context (work demands and needs)
		CP_05	The syllabus of that course is better than the one that is offered in other institutions to develop me professionally
		CP_06	The syllabus involves technological skills within my professional training
		CP_07	Academic subjects allow the application of knowledge in the professional field
		CP_08	Academic subjects allow me to develop my professional skills
		CP_09	Academic subjects develop problem-solving skills in the professional field
		CP_10	Academic subjects prepare me to be a professional capable of making decisions in the tourism field
		CP_11	In the academic subjects are developed practices or simulations that allow me to prepare myself better as a tourism professional
		CP_12	Academic subjects promote the efficient use of technological resources in professional activities and labors
	Student autonomy (SA)	SA_01	I am academically committed to my professional training
		SA_02	I am always looking for new ways or alternatives to improve my professional training
		SA_03	I am always willing to strengthen my professional training with the support of technological resources
		SA_04	I apply the acquired knowledge to work as a tourism professional
		SA_05	I have the initiative to strengthen my professional training with independent or self-study
Digital competence (DC)	Digital knowledge (DK)	DK_01	I have enough knowledge to manage the digital resources
		DK_02	I quickly understand the environments or usage logics used by the different digital media
		DK_03	I develop reflective thinking through digital media
		DK_04	I use my knowledge about digital resources to facilitate my activities, tasks or academic work, work, family, social, among others
		DK_05	I constantly evaluated my knowledge about the use of digital media
	Information management (IM)	IM_01	I locate useful information through Internet tools (search engines, web pages, and databases)
		IM_02	I get information from the Internet in real-time and anywhere
		IM_03	I evaluate the reliability of the information based on some criteria
		IM_04	I use responsibly the information, I obtain from digital media
	Individual digital communication (IDC)	IDC_01	I communicate effectively through digital media
		IDC_02	To communicate quickly, I prefer to use digital media
		IDC_03	I establish better communication channels when I take advantage of digital communities
	Collective digital communication (CDC)	CDC_01	I actively participate in online conversations or discussions by making valuable contributions to problem-solving
		CDC_02	By communicating through digital media, I am more productive in my tasks or activities
		CDC_03	Communication through digital media has made my network of social, academic and work contacts bigger
	Networked collaborative learning (NCL)	NCL_01	I actively participate in collaborative work using digital media
		NCL_02	I collaborate with others more effectively when I use digital media
		NCL_03	I collaborate with others to generate new things (ideas, knowledge, products, resources or content) through digital media
		NCL_04	I share other ideas, experiences, information or knowledge using digital media
		NCL_05	I contribute to the learning of my peers by sharing my knowledge through digital media
	Network leadership (NL)	NL_01	As a leader, I am always attentive to digital changes that may affect the work of my team
		NL_02	As a leader, I encourage my team to use digital resources
		NL_03	As a leader, I provide my team with digital facilities (access to information, resources and tools) for the fulfillment of goals
		NL_04	As a leader, I get the commitment of my team to work remotely using digital media
		NL_05	As a leader, I use digital media to solve the problems that arise within my team
		NL_06	As a leader, I motivate my team to be more and more competitive in the use of digital media
